# Staphylococcal enterotoxin B influences the DNA methylation pattern in nasal polyp tissue: a preliminary study

**DOI:** 10.1186/1710-1492-9-48

**Published:** 2013-12-16

**Authors:** Claudina A Pérez-Novo, Yuan Zhang, Simon Denil, Geert Trooskens, Tim De Meyer, Wim Van Criekinge, Paul Van Cauwenberge, Luo Zhang, Claus Bachert

**Affiliations:** 1 Upper Airways Research Laboratory, Department of Otorhinolaryngology, Ghent University Hospital, De Pintelaan 185, Ghent B-9000, Belgium; 2 Department of Otolaryngology, Head and Neck Surgery, Beijing Tongren Hospital, Capital Medical University, Beijing 100730, PR China; 3 Key Laboratory of Otolaryngology, Head and Neck Surgery (Ministry of Education of China), Beijing Institute of Otorhinolaryngology, Beijing 100005, PR China; 4 Department of Mathematical Modelling, Statistics and Bioinformatics, Faculty of Bioscience Engineering, Ghent University, Ghent, Belgium; 5 Karolinska Institutet, Division of ENT Diseases, CLINTEC, Stockholm, Sweden

**Keywords:** *Staphylococcus aureus* enterotoxin B, Chronic rhinosinusitis and nasal polyps, DNA methylation, MBD2, Whole genome methylation analysis, Hypermethylation

## Abstract

*Staphylococcal* enterotoxins may influence the pro-inflammatory pattern of chronic sinus diseases via epigenetic events. This work intended to investigate the potential of staphylococcal enterotoxin B (SEB) to induce changes in the DNA methylation pattern. Nasal polyp tissue explants were cultured in the presence and absence of SEB; genomic DNA was then isolated and used for whole genome methylation analysis. Results showed that SEB stimulation altered the methylation pattern of gene regions when compared with non stimulated tissue. Data enrichment analysis highlighted two genes: the IKBKB and STAT-5B, both playing a crucial role in T- cell maturation/activation and immune response.

## Background

*Staphylococcus aureus* enterotoxins acting as superantigens are known biological factors amplifying the pro-inflammatory patterns of upper airway inflammatory diseases, specifically chronic rhinosinusitis with nasal polyposis (CRSwNP) [1,2]. Recently, it has been demonstrated that bacterial infection and viral superantigens may lead to epigenetic deregulations affecting host cell functions [3]. This study aimed to investigate the potential of *S. aureus* enterotoxin B (SEB) to induce changes in the gene DNA methylation pattern in inflamed nasal tissue.

### Subjects and methods

**Figure 1:**
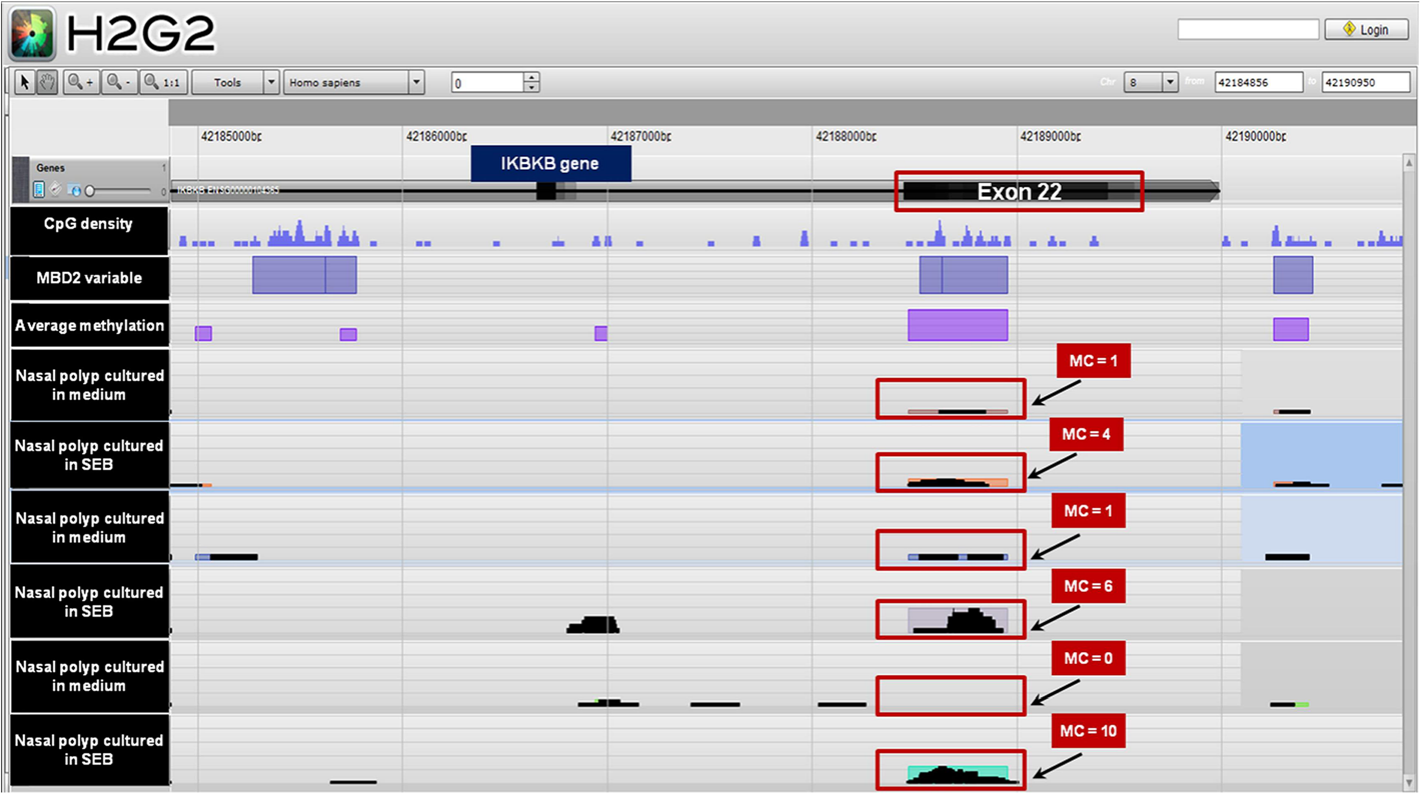
**Example of the visual representation of the results from MBD2 DNA methylation based analysis.** The figure shows the methylation cores (MC) for the differentially methylated region (exon 22) of the gene IKBKB on the genome browser "The Hitchhiker’s guide to the Genome" (http://www.biobix.be). The height of the black peaks shows the methylation level in that specific region in samples cultured in medium and with staphylococcal enterotoxin B (SEB).

A detailed description of the procedures followed in the study is provided in the Additional file 1. Briefly, nasal polyp tissues from 3 patients with chronic rhinosinusitis and nasal polyposis were fragmented and homogenized as described previously [4] and subsequently cultured during 24 h in the absence or presence of 0,5 μg/ml of SEB (Sigma-Aldrich, MO, United States). After stimulation, genomic DNA was isolated and used for a whole genome methyl-CpG-binding domain2 (MBD2)- based DNA methylation analysis [5]. The sequence reads obtained were then mapped using BOWTIE [6] and the data were summarized using a MethylCap kit specific “Map of the Human Methylome” (http://www.biobix.be) containing 1,518,879 potentially methylated sites termed methylation cores (MCs) as shown in Figure 1. Methylation was defined as the peak coverage in the MCs and was analyzed with the software package "R" version 2.11.1.

## Results

**Figure 2:**
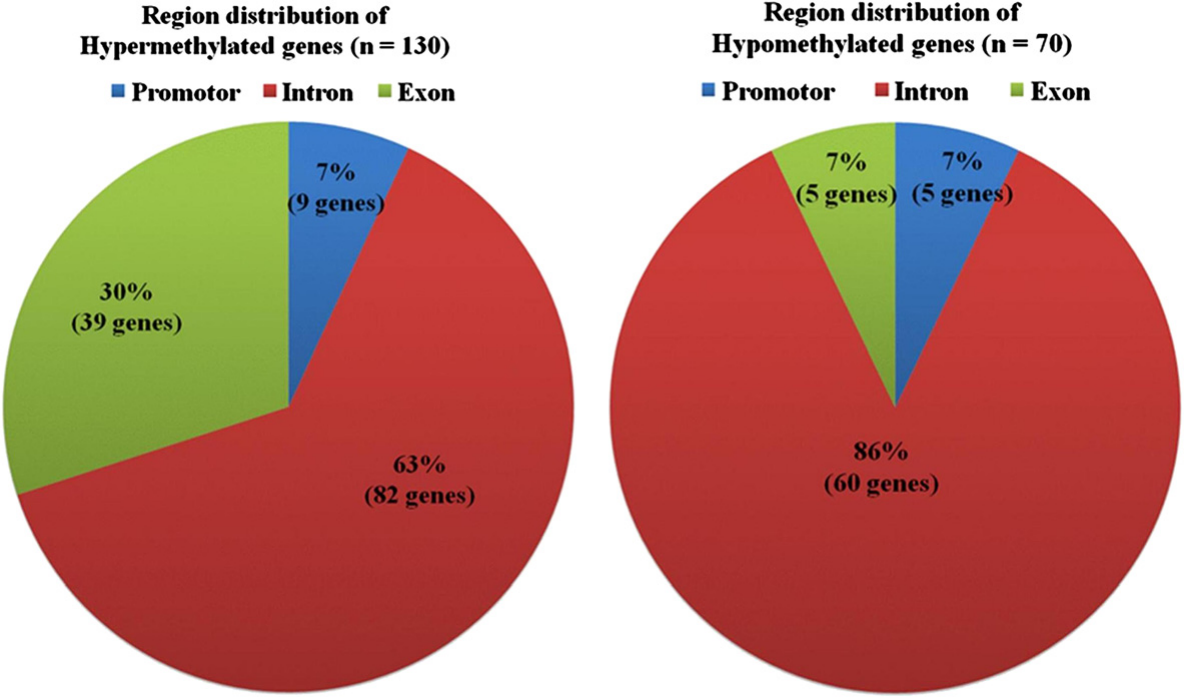
**Distribution of the genomic regions showing differential methylation cores.** The figure shows the percentage of genes showing different methylation cores in nasal polyp tissue cultures stimulated with *S. aureus* enterotoxin B (SEB) when compared with non-stimulated tissue. Most of the methylation changes occurred in intragenic regions (exons and introns) and in less extend at the promoter genes site.

A summary of the methylation data and analysis is provided in the repository file 1. In order to identify the genes which methylation status was affected by SEB stimulation, the obtained methylation cores (MCs) were ranked by “Likelihood Treatment” in descending order and an arbitrary "cut-off" was applied to select the 200 top differentially methylated genes. This ranking showed that stimulation with SEB mainly resulted in *de novo* hypermethylation (130 MCs) rather than in hypomethylation (70 MCs) and as expected, the methylation changes mainly occurred at intragenic regions (introns and exons) and to a lesser extend at the promoter or transcription start sites, as there were many more exonic and intronic MCs than promoter MCs in the entire map (Figure 2).

The 200 MCs primarily selected were then filtered using a “Likelihood Treatment” cut-off of 0.4 or more which translates to an estimated 40% probability that the MC is differentially methylated between samples treated or not with SEB. This cut-off value was used due to the low likelihood treatment values and low confidence obtained as result of the low coverage. This process provided a list of 43 genes exhibiting changes in the methylation state after 24 h culture with SEB (Table 1). From this list, 33 genes were hypermethylated while 10 genes showed hypomethylation. Three genes showed hypermethylations at promoter regions, and 18 and 12 genes at the intron and exon regions, respectively. Hypomethylation events were less frequent and they occurred at exonic regions in 9 genes, at introns in 1 gene and none at the promoter site (Table 1). Additionally, changes in the methylation status in other regions of these genes were also observed, but they did not pass the likelihood treatment cut-off due to low coverage; this may be solved in future studies as high coverage becomes affordable due to declining sequencing costs.

**Table 1:** Genes with different methylation status after stimulation with SEB

**Methylation status**	**Location**	**Gene**	**Chr**	**Likelihood**	**Ensemble accession**	**Methylation score TCM**	**Methylation score SEB**
Hypermethylation	Promoter	CTSLL2	10	0.599515	ENSG00000224036	1	7
		Y_RNA	6	0.595036	ENSG00000201555	1	8
		AC022026.3	10	0.589686	ENSG00000213731	0	6
	Intron	CHD5	1	0.887026	ENSG00000116254	0	8
		STAB2	12	0.644866	ENSG00000136011	1	8
		ROBO1	3	0.53597	ENSG00000169855	0	5
		AJAP1	1	0.512208	ENSG00000196581	1	6
		TTLL1	22	0.4962	ENSG00000100271	0	4
		GLT1D1	12	0.472979	ENSG00000151948	1	8
		MAD1L1	7	0.47049	ENSG00000002822	0	5
		HEATR5B	2	0.4673	ENSG00000008869	0	4
		LGMN	14	0.459486	ENSG00000100600	0	5
		FAM59A	18	0.458542	ENSG00000141441	0	4
		STAT5B	17	0.458148	ENSG00000173757	0	5
		NKD1	16	0.457063	ENSG00000140807	1	7
		SLC25A24	1	0.431776	ENSG00000085491	1	7
		AC073343.1	7	0.430122	ENSG00000228010	1	6
		TMEM138	11	0.412654	ENSG00000149483	1	6
		MPRIP	17	0.411674	ENSG00000133030	1	7
		GAA	17	0.40881	ENSG00000171298	1	8
		RFX3	9	0.407894	ENSG00000080298	1	6
	Exon	ADAMTS16	5	0.552893	ENSG00000145536	1	10
		IKBKB	8	0.533067	ENSG00000104365	1	7
		ZNF541	19	0.513317	ENSG00000118156	1	8
		KANK2	19	0.495541	ENSG00000197256	0	5
		CYBA	16	0.484142	ENSG00000051523	0	4
		UBE2I	16	0.471502	ENSG00000103275	1	6
		OLFM1	9	0.446344	ENSG00000130558	1	7
		MARK2	11	0.438781	ENSG00000072518	1	7
		CORO7	16	0.434694	ENSG00000103426	1	6
		KCNQ2	20	0.428704	ENSG00000075043	1	8
		ASAP1	8	0.414992	ENSG00000153317	1	6
		NOC2L	1	0.412654	ENSG00000188976	1	6
Hypomethylation	Intron	POLR3E	16	0.647331	ENSG00000058600	4	0
		VPS13B	8	0.576756	ENSG00000132549	4	0
		ANKRD13A	12	0.520639	ENSG00000076513	4	0
		ZBTB20	3	0.491524	ENSG00000181722	4	0
		AC087393.2	17	0.44588	ENSG00000233098	5	1
		ZDHHC1	16	0.440425	ENSG00000159714	3	0
		PDZD2	5	0.440425	ENSG00000133401	3	0
		DLGAP2	8	0.423913	ENSG00000198010	5	1
		NDST1	5	0.405485	ENSG00000070614	3	0
	Exon	AC016907.1	2	0.493142	ENSG00000233862	3	0

The table shows the nasal polyp tissue genes and locations undergoing methylation changes (hypermethylation and hypomethylation) with a likelihood treatment > 0.4) after stimulation with SEB. Methylation score refers to the average methylation of the 3 samples in each experimental group. TCM: tissue culture medium or no stimulated cells, SEB: cells stimulated with S. aureus enterotoxin B.

These 43 top ranking genes were then selected for enrichment analysis in the Reactome database using the overrepresentation pathway analysis [7]. This algorithm delivered a list of “Statistically over-represented pathways” which represents all Reactome pathways containing proteins from the input gene list. This analysis resulted in 17 pathways (Table 2) containing 6 potentially affected genes (STA5B, IKBKB, STAB2, NDST1, LGMN and CYBA). Based on previously published data regarding host-cellular immune responses to bacterial exotoxins we selected three main pathways (Table 3) containing the genes: STAT5B, IKBKB, POLR3 and LGMN. These genes regulate processes influencing the response of cells to superantigens according to the biological function obtained in UniProt and the Reactome databases (Table 3).

**Table 2:** Biological pathway analysis of the 43 top ranked genes showing differential methylation after simulation with SEB

***P-value***	**Number of genes mapping the pathway**	**Total number of genes in the pathway**	**Pathway identifier**	**Pathway name**	**Genes mapping to the pathway**
0,004	2	58	REACT_118823	Cytosolic sensors of pathogen-associated DNA	IKBKB, POLR3E
0,012	2	110	REACT_22232	Signaling by interleukins	STAT5B, IKBKB
0,013	2	115	REACT_6966	Toll-like receptors cascades	LGMN, IKBKB
0,015	2	120	REACT_121315	Glycosaminoglycan metabolism	STAB2, NDST1
0,015	2	120	REACT_147739	MPS IX - Natowicz syndrome	STAB2, NDST1
0,015	2	120	REACT_147853	Mucopolysaccharidoses	STAB2, NDST1
0,015	2	120	REACT_147788	MPS IIIB - Sanfilippo syndrome B	STAB2, NDST1
0,015	2	120	REACT_147719	MPS VI - Maroteaux-Lamy syndrome	STAB2, NDST1
0,015	2	120	REACT_147825	MPS IV - Morquio syndrome A	STAB2, NDST1
0,015	2	120	REACT_147860	MPS IIIC - Sanfilippo syndrome C	STAB2, NDST1
0,015	2	120	REACT_147759	MPS VII - Sly syndrome	STAB2, NDST1
0,015	2	120	REACT_147734	MPS II - Hunter syndrome	STAB2, NDST1
0,015	2	120	REACT_147857	MPS I - Hurler syndrome	STAB2, NDST1
0,015	2	120	REACT_147749	MPS IIID - Sanfilippo syndrome D	STAB2, NDST1
0,015	2	120	REACT_147753	MPS IIIA - Sanfilippo syndrome A	STAB2, NDST1
0,015	2	120	REACT_147798	MPS IV - Morquio syndrome B	STAB2, NDST1
0,045	4	915	REACT_116125	Disease	STAT5B, STAB2, NDST1, CYBA

All genes used in the analysis showed a likelihood of treatment related effect > 0,4. *P*-*value*: un-adjusted, not corrected for multiple testing, representing the probability (from hypergeometric test) of finding a given number or more genes in each pathway by chance.

**Table 3:** **Sub**-**pathways and biological functions of the most representative genes showing hyper**-**methylation after stimulation with SEB**

**Gene**	**UniProt**	**Pathway name**	**Sub-pathways**	**Biological function**
	**ID**	**(Reactome)**	**(Reactome)**	**(UniProt)**
**IKBKB**	O14920	Cytosolic sensors of pathogen-associated DNA	ZBP1 mediated induction of type I Interferons	Serine kinase that plays an essential role in the NF-kappa-B signaling pathway which is activated by multiple stimuli such as inflammatory cytokines, bacterial or viral products, DNA damages or other cellular stresses. It is involved in the transcriptional regulation of genes involved in immune response, growth control, or protection against apoptosis. May prevent the overproduction of inflammatory mediators since they exert a negative regulation on canonical IKKs.
		Adaptative immune response	TCR signaling	
		Signaling by interleukins	IL-1 signaling	
		Toll-Like receptors cascades	TLR2, TLR3, TLR5, TLR6, TLR7, TLR8, TLR9, TLR10	
**POLR3E**	Q9NVU0	Cytosolic sensors of pathogen-associated DNA	Transcription of microbial dsDNA to dsRNA	Plays a key role in sensing and limiting infection by intracellular bacteria and DNA viruses. Acts as nuclear and cytosolic DNA sensor involved in innate immune response. Can sense non-self dsDNA that serves as template for transcription into dsRNA. The non-self RNA polymerase III transcripts, such as Epstein-Barr virus-encoded RNAs (EBERs) induce type I interferon and NF- Kappa-B through the RIG-I pathway.
**STAT5B**	P51692	Signaling by interleukins	Signaling of IL-2, IL-3, IL-5, IL-7 and GMCSF	Carries out a dual function: signal transduction and activation of transcription. Mediates cellular responses to the cytokine KITLG/SCF and other growth factors. Binds to the GAS element and activates PRL-induced transcription.
**LGMN**	Q99538	Toll-Like receptors cascades	Trafficking and processing of endosomal TLR	It is involved in the processing of proteins for MHC class II antigen presentation in the lysosomal/endosomal system.

The genes for this analysis were selected from the Reactome over-representation pathway analysis.

This study did not include healthy nasal mucosa. We specifically investigated whether *S. aureus* enterotoxin B might influence the gene DNA methylation pattern in inflamed (nasal polyp) tissue without studying the effects of the diseased status itself. Indeed, validation experiments including a larger number of samples as well as samples from control (healthy) tissue are warrented in light of these preliminary results. Also we could not preclude effects of other staphylococcal superantigens or superantigens from other germs as the nose is a hotspot of micro-organism activity [8]. However, although methylation differences due to other enterotoxins are a distinct possibility, this should not affect the results as both SEB treated and untreated cells originated from the same patients. Only if significant concentrations of other enterotoxins were present in all 3 patients might this confound the results. In conclusion, these preliminary findings suggest DNA methylation as a possible mechanism by which superantigens may regulate immune function in the nasal mucosa.

## Competing interests

We, the authors declare that:

We have not received any reimbursements, fees, funding, or salary from an organization that may in any way gain or lose financially (now or in the future) from the publication of this manuscript. We do not hold any stocks or shares in an organization that may in any way gain or lose financially (now or in the future) from the publication of this manuscript. We do not hold and we are not currently applying for any patent relating to the content of the manuscript. We have not received reimbursements, fees, funding, or salary from an organization that holds or has applied for patents relating to the content of this manuscript.

We have no "non-financial" competing interests such as political, personal, religious, ideological, academic, intellectual, commercial etc. to declare in relation to this manuscript.

## Authors’ contributions

CAPN contributed with the design of the experiments, sample collection, stimulation experiments, data analysis and writing of the manuscript. YZ contributed with the data analysis, writing and revision of the manuscript. SD performed the MBD2 differential methylation analysis and contributed with the writing and revision of the manuscript. GT performed the MBD2 peak-calling, data visualization and base calling analysis. TDM contributed with the design of the algorithm to construct the methylome map (i.e. determine methylation core locations). WvC organized and supervised the sequencing experiments and data analysis. PvC contributed with the design of the experiments and the revision of the manuscript. LZ contributed with the writing and revision of the manuscript. CB contributed with the design of experiments, sample collection and with the writing and revision of the manuscript. All authors read and approved the final manuscript.

## Supplementary Material

Additional file 1 Description of the data: These files include more detailed information about the patient’s characteristics, methodologies used and results obtained in the study. Click here for file
