# Supplementary material for: Staphylococcal enterotoxin B influences the DNA methylation pattern in nasal polyp tissue: a preliminary study
Source: Allergy Asthma Clin Immunol. 2013 Dec 16;9(1):48. doi: 10.1186/1710-1492-9-48 (PMC3867657; doi:10.1186/1710-1492-9-48)
Supplement: Additional file 1 — Description of the data: These files include more detailed information about the patient’s characteristics, methodologies used and results obtained in the study. [file 1710-1492-9-48-S1.doc]

**ONLINE SUPPLEMENTARY INFORMATION**

**PATIENTS**

The study was performed in nasal polyp tissue obtained from 3 patients with chronic rhinosinusitis and nasal polyposis (CRSwNP) during scheduled functional endoscopic sinus surgery performed at the Department of Otorhinolaryngology at the Ghent University Hospital. Patients had no allergy, asthma or hypersensitivity to aspirin or nonsteroidal anti-inflammatory drugs. The study was approved by the local Research Ethics Committee and written informed consent was obtained from each patient.

**METHODOLOGY**

Nasal polyp tissues were fragmented and homogenized and cultured during 24 hours in the absence or presence of 0,5 µg/ml of *S. aureus* enterotoxin B (Sigma Aldrich, MO, United States). After stimulation, genomic DNA was isolated and sheared in a Covaris S220 ultrasonicator (Covaris, MA, USA) that was set to obtain an average fragment size of 200 base pairs (bp). Subsequently 200 ng of fragmented DNA were precipitated with the methyl-CpG-binding domain2 (MBD2)- MethylCap kit (Diagenode, Liège, Belgium) following manufacturers' protocol. The precipitated DNA was then sequenced in an Illumina GAIIx (run performed by NXTGNT, Gent, Belgium) with the DNA paired-end sequencing kit (multiplexed) 2 x 45 bp and 7 bp's index. The sequence reads were then mapped using BOWTIE ([http://bowtie.cbcb.umd.edu](http://bowtie.cbcb.umd.edu/)) and data were summarized using a MethylCap kit specific “Map of the Human Methylome” ([www.biobix.be/mhm](http://www.biobix.be/mhm)) containing 1,518,879 potentially methylated sites termed methylation cores (MCs). Methylation was defined as the peak coverage in the MCs and was analyzed with the software package "R" version 2.11.1.

**RESULTS**

Sequencing generated 2.943,199 to 5.671,716 mapped reads per sample. Initial analysis was limited to those regions located within annotated genes or their promoter region (up to 2000 bp upstream, Ensemble v56) and which had at least 3 mapped reads summed over all 6 samples (768.475 MCs total). Of the total number of MCs, 59280 are in known promoter regions (-2000 bp to 500 bp) or exon 1. Data analysis was performed using the R package “baySeq” [1], designed to assign the plausibility or "likelihoods" to count data (negative binomial distribution). The sample size was set to be 5 times the number of variables. Standard settings were used where possible, priors were estimated by sampling all variables and posteriors were estimated by bootstrapping 3 times. The counts were normalized using the Trimmed Mean of *M*-values method using standard settings as implemented in the “edgeR” package [2]. This approach allowed us to evaluate three possible models: no differential methylation, donor specific methylation or differential methylation between samples cultured with or without SEB.

**REFERENCES**

1. Hardcastle TJ, Kelly KA: **baySeq: empirical Bayesian methods for identifying differential expression in sequence count data.** *BMC Bioinformatics* 2010, **11:**422.

2. Robinson MD, Oshlack A: **A scaling normalization method for differential expression analysis of RNA-seq data.** *Genome Biol* 2010, **11:**R25.
